# Metabolomic Analysis by Nuclear Magnetic Resonance Spectroscopy as a New Approach to Understanding Inflammation and Monitoring of Pharmacological Therapy in Children and Young Adults With Cystic Fibrosis

**DOI:** 10.3389/fphar.2018.00595

**Published:** 2018-06-18

**Authors:** Paolo Montuschi, Vincenzina Lucidi, Debora Paris, Enza Montemitro, Rugia Shohreh, Nadia Mores, Dominique Melck, Giuseppe Santini, Fabio Majo, Andrea Motta

**Affiliations:** ^1^Department of Pharmacology, Faculty of Medicine, Catholic University of the Sacred Heart, Rome, Italy; ^2^Pharmacology Unit, Fondazione Policlinico Universitario Agostino Gemelli IRCCS, Rome, Italy; ^3^Cystic Fibrosis Unit, Ospedale Pediatrico Bambino Gesù, Rome, Italy; ^4^Institute of Biomolecular Chemistry, Italian National Research Council, Pozzuoli, Italy; ^5^Department of Drug Sciences, Faculty of Pharmacy, University “G. d’Annunzio” Chieti-Pescara, Chieti, Italy

**Keywords:** NMR spectroscopy, metabolomics, isoprostanes, oxidative stress, cystic fibrosis, pharmacotherapy, azithromycin

## Abstract

15-F_2t_-Isoprostane, a reliable biomarker of oxidative stress, has been found elevated in exhaled breath condensate (EBC), a non-invasive technique for sampling of airway secretions, in patients with cystic fibrosis (CF). Azithromycin has antioxidant properties in experimental models of CF, but its effects on oxidative stress in CF patients are largely unknown. Primary objective of this pilot, proof-of-concept, prospective, parallel group, pharmacological study, was investigating the potential antioxidant effects of azithromycin in CF patients as reflected by EBC 15-F_2t_-isoprostane. Secondary objectives included studying the effect of azithromycin on EBC and serum metabolic profiles, and on serum 15-F_2t_-isoprostane. In CF patients who were on maintenance treatment with oral vitamin E (200 UI once daily), treatment with oral azithromycin (250 or 500 mg depending on body weight) plus vitamin E (400 UI once daily) (group A) (*n* = 24) or oral vitamin E alone (400 UI once daily) (group B) (*n* = 21) was not associated with changes in EBC 15-F_2t_-isoprostane concentrations compared with baseline values after 8–weeks treatment or 2 weeks after treatment suspension. There was no between-group difference in post-treatment EBC 15-F_2t_-isoprostane. Likewise, no within- or between-group differences in serum 15-F_2t_-isoprostane concentrations were observed in either study group. NMR spectroscopy-based metabolomics of EBC shows that suspension of both azithromycin plus vitamin E and vitamin E alone has a striking effect on metabolic profiles in EBC. Between-group comparisons show that EBC metabolite distribution after treatment and 2 weeks after treatment suspension is different. Quantitative differences in ethanol, saturated fatty acids, acetate, acetoin/acetone, and methanol are responsible for these differences. Our study was unable to show antioxidant effect of azithromycin as add-on treatment with doubling the dose of oral vitamin E as reflected by 15-F_2t_-isoprostane concentrations in EBC. Add-on therapy with azithromycin itself does not induce EBC metabolite changes, but its suspension is associated with EBC metabolic profiles that are different from those observed after vitamin E suspension. The pathophysiological and therapeutic implications of these findings in patients with stable CF are unknown and require further research. Preliminary data suggest that EBC NMR-based metabolomics might be used for assessing the effects of pharmacological treatment suspension in stable CF patients.

## Introduction

Airway inflammation and oxidative stress have a central role in the pathophysiology of cystic fibrosis (CF) (Brown and Kelly, 1994; Cantin et al., 2006, 2007; Elizur et al., 2008; Elborn, 2016). Assessment of respiratory inflammation is relevant for management of CF patients. Exhaled breath condensate (EBC), a non-invasive method for sampling airway secretions, which is likely to reflect respiratory inflammation (Montuschi, 2002; Effros et al., 2005; Montuschi, 2007; Horváth et al., 2017), is potentially useful for assessing and monitoring of airway inflammation (Montuschi, 2002; Montuschi, 2007). Metabolomics, the study of molecules generated by metabolic pathways, defines the metabolic phenotype (“metabotype”), offers a source of novel biomarkers that have relevant applications to pharmaceutical development and patient management (Rochfort, 2005; Kaddurah-Daouk and Krishnan, 2009; Puchades-Carrasco and Pineda-Lucena, 2015; Kohler et al., 2017), can be used for assessing oxidative stress non-invasively, and has been successfully applied to EBC analysis in patients with CF (Montuschi et al., 2012, 2014). The development of effective anti-inflammatory/antioxidant therapies has been limited by the lack of sensitive outcome measures (Elizur et al., 2008) and non-invasive techniques for assessing lung inflammation/oxidative stress. Very few clinical prospective studies incorporated measures of oxidative stress. 15-F_2t_-Isoprostane, a reliable biomarker of oxidative stress (Morrow et al., 1990; Montuschi et al., 2004, 2007), is elevated in EBC in stable CF patients (Montuschi et al., 2000; Lucidi et al., 2008) and, to a greater extent, in unstable CF patients (Lucidi et al., 2008). Urinary excretion of 15-F_2t_-isoprostane has been found elevated in CF patients compared with healthy subjects (Ciabattoni et al., 2000). Azithromycin has antioxidant and immunomodulatory properties in experimental models of CF *in vitro* and *in vivo* (Cigana et al., 2006; Bergamini et al., 2009; Altenburg et al., 2011; Stellari et al., 2015; Leal et al., 2016). In CF patients, azithromycin has been reported to reduce serum biomarkers of inflammation (Wolter et al., 2002; Steinkamp et al., 2008), but its effects on oxidative stress are largely unknown. Improvement in respiratory function and reduction in pulmonary exacerbation rate have been shown after 6-month treatment with azithromycin (Southern et al., 2012). International guidelines support a role of azithromycin in the maintenance treatment of CF patients (Flume et al., 2007; Mogayzel et al., 2013).

Primary objective of this study was investigating the potential antioxidant effect of azithromycin in patients with CF as reflected by 15-F_2t_-isoprostane concentrations in EBC. Secondary objectives included studying the effect of this drug on EBC and serum metabolites detected by NMR spectroscopy. In particular, we aimed at obtaining information on the potential additive or synergistic antioxidant effects of azithromycin/vitamin E combination over vitamin E alone as assessed by measuring EBC and serum 15-F_2t_-isoprostane concentrations in patients with CF. This study was also undertaken to gain data on the effects of these therapies on the metabolomics profiles in EBC and serum.

## Materials and Methods

### Study Subjects

#### Inclusion Criteria

Forty-five males or females (age 6–20 years) who had stable CF on the basis of clinical, radiological, and genotypic characteristics consistent with CF and an abnormal sweat test (sweat chloride ≥ 60 mmol/L) (Stern, 1997) were recruited and completed the study. Subjects were never smokers, including no use of smokeless tobacco products. Patients had clinically stable CF with no change in cough or shortness of breath, no requirement for oral or intravenous antibiotic medication in the previous 4 weeks before study entry and no significant change in spirometry in the last 4 weeks. Patients were on a constant dose of oral vitamin E [200 UI/daily (=200 mg all-rac-alfa tocopheryl acetate = 225 mg dl-alfatocopheryl acetate or 134 mg RRR-alfa-tocopherol) as tablet] for at least 12 weeks.

#### Exclusion Criteria

Patients who underwent any major surgical procedure in the previous 4 weeks, participated in a clinical trial involving an investigational or marketed drug in the previous 4 weeks, had, in addition to CF, any active, acute or chronic pulmonary disorder documented by history or physical examination, had upper respiratory infections in the previous 3 weeks, or had unstable CF with pulmonary exacerbation were excluded. Pulmonary exacerbation was defined by the presence of at least two of the following signs: fever, more frequent coughing, increased sputum volume, loss of appetite, weight loss, absence from school or work due to illness, symptoms of upper respiratory tract infection (Ramsey et al., 1999).

Apart from CF, patients who had a clinically significant, active disease of the gastrointestinal, cardiovascular, hepatic, neurological, renal, genitourinary, or hematological systems, or uncontrolled hypertension (>160/95), or an immunodeficiency, or an autoimmune disorder, had a history of any illness that could be immediately life threatening (ventricular arrhythmia, neoplasia, incompletely cured or treated in the last 3 months, ‘brittle’ diabetes mellitus), or would pose restriction on participation in the study, or were hypersensitive to azithromycin or components in the pharmaceutical formulation were excluded.

Treatment with the following medications was not allowed: oral, intravenous, intramuscular, intra-articular or inhaled corticosteroids in the previous 4 weeks; other antioxidants, apart from vitamin E, and macrolides in the previous 2 weeks before visit 1.

### Experimental Plan and Study Design

This was a pilot, proof-of-concept, pharmacological, interventional, randomized, open label, parallel group study in which two groups of children and adolescents (age 6–20 years) with stable CF were treated with either a combination of azithromycin and vitamin E (group A) or vitamin E alone (group B).

Study design includes: (1) a run-in phase (1 week); (2) a treatment phase (8 weeks); a run-out phase (2 weeks). Study duration was 11 weeks and included four visits. EBC collection was performed at each visit for measurement of 15-F_2t_-isoprostane and C reactive protein concentrations and NMR-based metabolomics. Whole blood sampling was performed at each visit for measurement of 15-F_2t_-isoprostane and C reactive protein concentrations and NMR-based metabolomics in serum, and measurement of plasma vitamin E at visit 2 and 3. Pulmonary function tests were performed at each visit. Chest X ray was performed at visit 2 and 3.

**FIGURE 1:**
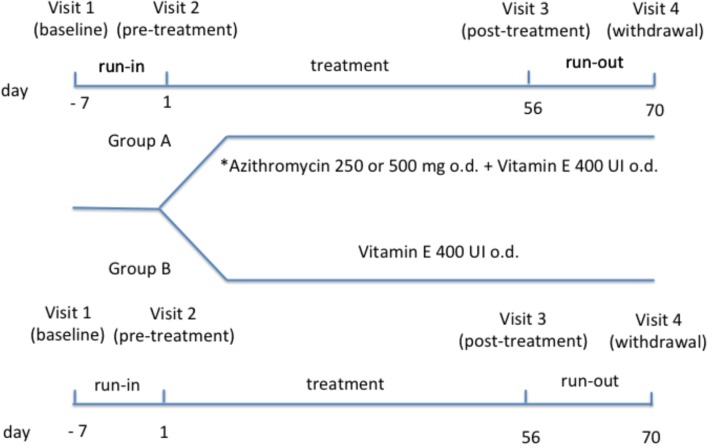
Study design. ^∗^Daily dose of azithromycin was 250 mg in subjects with body weight < 40 kg or 500 mg in subjects with body weight ≥ 40 kg.

After a baseline visit (visit 1) (day -7) and 1-week run-in period, patients who were on a constant dose of oral vitamin E [200 UI/daily (=200 mg all-rac-alfa-tocopheryl acetate = 225 mg dl-alfatocopheryl acetate or 134 mg RRR-alfa-tocopherol as tablet) for at least 12 weeks] were randomized to (1) a combination of azithromycin [250 mg (weight < 40 kg) or 500 mg (weight ≥ 40 kg) once daily as tablet] (Equi et al., 2002) and oral vitamin E [400 UI/daily (=400 mg all-rac-alfa-tocopheryl acetate = 450 mg dl-alfatocopheryl acetate or 268 mg RRR-alfa-tocopherol) as tablet] (day 1) (group A) or (2) vitamin E alone at the same dose (group B) (pre-treatment visit 2) (**Figure 1**). Twenty-four patients were included in group A and 21 patients were included in group B.

Subjects were randomly allocated to study groups by simple randomization using computer-generated random numbers (Suresh, 2011).

Duration of treatment phase was 8 weeks. After treatment phase (visit 3) (day 56) and after 2 weeks from drug withdrawal (visit 4) (day 70), all interventions/procedures were repeated. Patients were recruited and visits were performed at the Cystic Fibrosis Unit, Ospedale Pediatrico Bambino Gesù, Rome, Italy. Written informed consent was obtained from young adults or child’s parents with assent from the child. The study (number 333/2009) was approved by the Ethics Committee of Ospedale Pediatrico Bambino Gesù, Rome, Italy.

### Patient Recruitment

Seventy-three patients with CF attended the outpatient clinic of Cystic Fibrosis Unit, Ospedale Pediatrico Bambino Gesù, Rome, Italy, for possible inclusion into the present study. Nineteen patients were excluded as they did not meet the inclusion criteria. Fifty-four patients have been enrolled in this study. Nine patients were excluded from the study after baseline visit (6 patients due to CF exacerbation, 2 due to non-compliance, one due to consent withdrawal). Forty-five patients (24 in group A and 21 in group B) completed the study. Their characteristics are shown in **Table 1**.

**Table 1:** Subject characteristics^∗^.

	CF patients Group A	CF patients Group B	*P*-value
*n*	24	21	
Age, year	18 ± 1.8	18 ± 1.1	0.97
Sex, F/M	13/11	8/13	0.37
**Bacterial infection**		
*Pseudomonas aeruginosa*^#^	6	4	0.73
*Pseudomonas aeruginosa + Aspergillus*	1	0	1.0
*Pseudomonas aeruginosa + Stenotrophomonas maltophilia*	0	2	0.21
*Pseudomonas aeruginosa + Staphylococcus aureus + Stenotrophomonas maltophilia + Achromobacter xylosoxidans*	1	0	1.0
*Pseudomonas aeruginosa + Staphylococcus aureus + Stenotrophomonas maltophilia + Aspergillus*	1	0	1.0
*Pseudomonas aeruginosa + Staphylococcus aureus + Aspergillus*	1	0	1.0
*Pseudomonas aeruginosa + Staphylococcus aureus + Mycobacteria*	0	1	0.47
*Pseudomonas aeruginosa + Staphylococcus aureus + Aspergillus + Achromobacter xylosoxidans*	0	1	0.47
*Pseudomonas aeruginosa + Achromobacter xylosoxidans*	1	0	1.0
*Staphylococcus aureus*^§^	5	3	0.71
*Staphylococcus aureus + Pseudomonas aeruginosa*	4	4	1.0
*Staphylococcus aureus + Aspergillus*	1	1	1.0
*Staphylococcus aureus + Stenotrophomonas maltophilia + Aspergillus*	1	1	1.0
*Staphylococcus aureus + Stenotrophomonas maltophilia*	0	1	0.47
*Staphylococcus aureus + Stenotrophomonas maltophilia + Mycobacteria*	1	0	1.0
*Staphylococcus aureus + Pseudomonas aeruginosa + Stenotrophomonas maltophilia*	1	2	0.59
*Staphylococcus aureus + Pseudomonas aeruginosa + Stenotrophomonas maltophilia + Serratia + Aspergillus*	0	1	0.47
*Burkholderia cepacia*	0	0	1.0
**CFTR genotype**		
ΔF508/ΔF508	6	7	0.74
ΔF508/other	8	10	0.37
Other/other	10	4	0.12
**Pharmacological treatment**		
ICS (y/n)	0/24	0/21	1.0
Short-acting β_2_-agonists (y/n)	14/10	17/4	0.12
Inhaled tobramycin (y/n)	14/10	13/8	1.0
Inhaled colistin (y/n)	5/19	3/18	0.71
Recombinant human DNase (y/n)	9/15	13/8	0.14

*CF, cystic fibrosis; CFTR, cystic fibrosis transmembrane conductance regulator; CRP, C reactive protein; ICS, inhaled corticosteroids. ^∗^Data are expressed as n or mean ± SEM. Unpaired t test or Fisher’s exact test were used for comparing groups. Significance was defined as a value of P < 0.05. ^#^Eleven out of 24 CF patients in group A and 8 out of 21 CF patients in group B had a positive Pseudomonas aeruginosa sputum culture alone or combined with other microorganisms. P-value related to comparison between CF patients in groups A and B with positive or negative Pseudomonas aeruginosa sputum culture is 0.76. ^§^ Thirteen out of 24 CF patients in group A and 13 out of 21 CF patients in group B had a positive Staphylococcus aureus sputum culture alone or combined with other microorganisms. P-value related to comparison between CF patients in groups A and B with positive or negative Staphylococcus aureus sputum culture is 0.76. Patients with CF with concomitant allergy and/or asthma were excluded from the study.*

### Outcome Measures

15-F_2t_-isoprostane concentrations in EBC was the primary outcome measure. Secondary outcome measures included metabolomics of EBC, metabolomics of serum, 15-F_2t_-isoprostane concentrations in serum, CRP concentrations in EBC, CRP concentrations in serum, forced expiratory volume in one second (FEV_1_), forced vital capacity (FVC), and FEV_1_/FVC ratio.

### EBC Sampling

EBC was collected using a condensing chamber (Ecoscreen; Jaeger; Hoechberg, Germany) (Montuschi and Barnes, 2002). Subjects were instructed to breath tidally through a mouthpiece connected to the condenser for 15 min. EBC samples were stored at -80°C before metabolomic analysis with NMR spectroscopy and 15-F_2t_-isoprostane measurement. Salivary contamination was excluded by comparing metabolomic profiles of EBC and saliva identified with NMR spectroscopy (Motta et al., 2012). NMR spectroscopy showed that the pattern of metabolites in EBC was clearly different from that observed in saliva. When EBC samples were contaminated on purpose with saliva, the pattern of metabolites in these EBC samples was similar to that observed in saliva samples (Motta et al., 2012).

### NMR-Based Metabolomics of EBC and Serum, Pattern Recognition and Statistical Analysis

One-dimensional (1D) spectra of EBC were recorded on a Bruker Avance III spectrometer operating at a frequency of 600.13 MHz (^1^H), equipped with a TCI CryoProbe^TM^ fitted with a gradient along the *Z*-axis, at a probe temperature of 27°C (Montuschi et al., 2012). To eliminate the water signal, we used the excitation sculpting pulse sequence to suppress the water resonance (Hwang and Shaka, 1995). For signal identification, we relied upon homonuclear and heteronuclear two-dimensional (2D) experiments, namely, clean Total Correlation Spectroscopy (TOCSY) (Griesinger et al., 1988), and ^1^H-^13^C Heteronuclear Single Quantum Coherence (HSQC) spectrum (Kay et al., 1992). 1D Spectra were referred to 0.1 mM sodium trimethylsilylpropionate (TSP), assumed to resonate at δ = 0.00 ppm, while 2D spectra were referred to the lactate doublet (βCH_3_) resonating at 1.33 ppm for ^1^H, and at 20.76 ppm for ^13^C.

We used multivariate analysis to identify hidden phenomena and trends in ensembles of spectra generated by specific metabolites. Proton NMR spectra were automatically data reduced to 400 integral segments (“buckets”), each of 0.02 ppm, using the AMIX 3.6 software package (Bruker Biospin GmbH, Rheinstetten, Germany), between the 0.10–8.60 ppm region. The residual water resonance region (5.20–4.50 ppm) was excluded, and each integrated region was normalized to the total spectrum area to avoid possible signal variation due to sample dilution.

The data were imported into SIMCA-P+14 package (Soft Independent Modeling of Class Analogy; Umetrics, Umeå, Sweden), and pre-processed with Pareto scaling to account for the difference in NMR signals’ intensity. The model quality was evaluated by using the goodness-of-fit parameter (*R*^2^) and the goodness-of-prediction parameter (*Q*^2^) (Eriksson et al., 2013).

Principal component analysis (PCA) was first applied to detect EBC metabolites trends and clusterings in an unsupervised (i.e., no prior group knowledge is used in the calculation) manner (Bishop, 2006; Worley and Powers, 2013). However, to better identify clusterings, we tested both orthogonal projections to latent structures (OPLS) and orthogonal signal correction (OSC) routines together with the partial least-squares projections to latent structures-discriminant analysis (PLS-DA) to verify data fitting and possible data overfitting, which was excluded (Bishop, 2006; Worley and Powers, 2013). The obtained OPLS model turned out to be improved in terms of both predictive and interpretive ability. A permutation test (*n* = 300) was carried out to assess possible overfit of the model.

Statistical significance for selected metabolites was determined by parametric (Student’s *t*) or non-parametric Mann–Whitney *U*) tests according to the results of normality test performed to evaluate each distribution (Shapiro–Wilk, Kolmogorov–Smirnov test). *P*-values < 0.05 were considered as statistically significant.

### Measurement of 15-F_2t_-Isoprostane in EBC

15-F_2t_-isoprostane concentrations in EBC were measured with a specific radioimmunoassay (RIA) that was developed in our laboratory (Wang et al., 1995; Montuschi et al., 2003a). Specificity for 15-F_2t_-isoprostane RIA used in this study was previously confirmed by RP-HPLC (Montuschi et al., 2003b) and gas-chromatography/mass spectrometry (GC/MS) (Wang et al., 1995). Day-to-day reproducibility for 15-F_2t_-isoprostane measurements was previously assessed in 20 healthy subjects in a randomized design in which three EBC samples were collected on days 1, 3, and 7 (Montuschi et al., 2003a). Day-to-day reproducibility, expressed as intraclass correlation coefficient, was 0.95 (Montuschi et al., 2003a).

### Pulmonary Function Testing

Forced expiratory volume in 1 s (FEV_1_) and forced vital capacity (FVC) were measured by spirometry (Quark PFT2; Cosmed; Rome, Italy) and the best of three maneuvers, expressed as percentage of predicted values, was chosen.

### Measurement of CRP in EBC and Serum

One ml of whole blood was drawn for CRP measurement in serum. CRP was measured in EBC and serum with Tina-quant CRP particle-enhanced immunoturbidimetric method, an automated high sensitive CRP method, performed using a COBAS INTEGRA 400 analyzer (Roche Diagnostics; Basel, Switzerland). The analytic measurement range is 0–160 mg/l with automatic dilution for results up to 1600 mg/l.

### Measurement of Plasma Vitamin E

Vitamin E concentrations were determined in 200 μl plasma samples by reversed-phase high performance liquid chromatography (RP-HPLC) in isocratic conditions at 0.6 ml/min flow rate with UV detection at 295 nm according to manufacturer instructions (Bio-Rad, Hercules, CA, United States).

### Sample Size

15-F_2t_-isoprostane concentrations in EBC after 8-week treatment with azithromycin were considered as the primary outcome. Sample size was calculated on the basis of a previous study (Lucidi et al., 2008) and was estimated to be 23 subjects per group (results for single sided), after having considered a SD of 6.4 pg/ml, a dropout of 20%, and identified the minimal difference of biological significance (6.1 pg/ml corresponding to 20% reduction of mean 15-F_2t_-isoprostane concentrations in EBC in the azithromycin group) with a power of 90% (α = 5%, β = 10%).

### Statistical Analysis

Data are expressed as medians and interquartile range (25 to 75 percentiles) or mean ± SEM or mean ± SD depending on data distribution. Newman–Keul repeated-measures analysis of variance or Friedman repeated-measures analysis of variance were used to compare values within each treatment arm for normally distributed and non-parametric data, respectively. Unpaired *t*-tests or Mann–Whitney test were used for between-group treatment comparisons for normally distributed and non-parametric data, respectively.

Correlation is expressed either as Spearman’s coefficient or Pearson’s coefficient on the basis of data distribution. Significance is defined at *P* < 0.05.

To warrant a blind approach to analytical procedures, the Principal Investigator and Dr. Andrea Motta, who were responsible for measurement of 15-F_2t_-isoprostane and metabolites in EBC and serum samples, did not know the type of treatment in groups A and B.

## Results

Demographic characteristics of patients with CF included in groups A and B are shown in **Table 1**.

The two study groups were similar regarding gender, age, sputum culture microbiology, Cystic Fibrosis Transmembrane Conductance Regulator (CFTR) genotype, and pharmacological treatment (**Table 1**).

Eleven out of 24 CF patients in group A (46%) and 8 out of 21 CF patients in group B (33%) had a positive *Pseudomonas aeruginosa* sputum culture alone or combined with other microorganisms (**Table 1**). Thirteen out of 24 CF patients in group A (54%) and 13 out of 21 CF patients in group B (62%) had a positive *Staphylococcus aureus* sputum culture alone or combined with other microorganisms (**Table 1**). Six out of 24 CF patients in group A (25%) and 7 out of 21 CF patients in group B (33%) were homozygous for the ΔF508 CFTR genotype (**Table 1**).

### Measurement of 15-F_2t_-Isoprostane in Exhaled Breath Condensate

In group A, 80 EBC samples were analyzed. Sixteen samples were missing. However, at least a pre-treatment (visit 2) and post-treatment (visit 3) EBC sample was collected from each patient. 15-F_2t_-Isoprostane concentrations, ranging from 83.6 to 598.8 pg/ml, were detected in all EBC samples. These values are well above the detection limit of the analytical assay. Due to missing samples at visit 1 and visit 4, paired *t*-test instead of analysis of variance for repeated measures was used for assessing within group treatment effect.

Pre-treatment EBC 15-F_2t_-isoprostane values in study groups were similar (group A, 325.0 ± 115.2 pg/ml, mean ± SD; group B, 329.1 ± 150.7 pg/ml, *P* = 0.92).

Compared with pre-treatment values observed in CF patients who were on a constant dose of oral vitamin E (200 UI/daily), there was no difference in 15-F_2t_-isoprostane concentrations in EBC after 8-week treatment with the combination of a higher dose of oral vitamin E (400 UI/daily) and azithromycin (250 or 500 mg once daily depending on body weight) (primary outcome) indicating no additional effect on oxidative stress as reflected by EBC 15-F_2t_-isoprostane (**Table 2**).

**Table 2:** 15-F_2t_-Isoprostane concentrations in EBC in groups A and B^∗^.

Group A (*n* = 24)^#^		
	
	Visit 1 (baseline) (*n* = 19)^§^	Visit 2 (pre-treatment) (*n* = 24)^§^	Visit 3 (post-treatment) (*n* = 24)^§^	Visit 4 (treatment withdrawal) (*n* = 13)^§^	*P*-value°
15-F_2t_-IsoP (pg/ml)	326.3 ± 112.8	325.0 ± 115.2	314.9 ± 131.7	317.1 ± 128.8	0.79

**Group B (*n* = 21)^#^**		
	
	**Visit 1 (baseline) (*n* = 15)^§^**	**Visit 2 (pre-treatment) (*n* = 21)^§^**	**Visit 3 (post-treatment) (*n* = 21)^§^**	**Visit 4 (treatment withdrawal) (*n* = 10)^§^**	***P*-value°**

15-F_2t_-IsoP (pg/ml)	309.3 ± 182.0	329.1 ± 150.7	343.1 ± 243.9	245.5 ± 157.1	0.81

*^∗^Data are expressed as mean ± SD as they were normally distributed after log transformation. Due to missing samples at visits 1 and 4, within group effect of treatment was assessed by paired t-test applied to pre-treatment values and post-treatment values. Between-group values, assessed by unpaired t-test, at visit 1 (P = 0.74), visit 2 (P = 0.92), visit 3 (P = 0.63), and visit 4 (P = 0.23) were similar. Significance was defined as a value of P < 0.05. ^#^Number of CF subjects included in the study group. ^§^Number of samples analyzed per visit. °P-value is related to comparison between visits 2 and 3. 15-F_*2t*_-IsoP, 15-F_*2t*_-isoprostane.*

In group B, 67 EBC samples were analyzed. Seventeen samples were missing. However, at least a pre-treatment (visit 2) and post-treatment (visit 3) EBC sample was collected from each patient. 15-F_2t_-Isoprostane concentrations were detected in EBC in all, but one, samples. EBC 15-F_2t_-isoprostane concentrations ranged from 92.1 to 974.2 pg/ml, values which are well above the detection limit of the analytical assay.

Due to missing samples at visit 1 and visit 4, paired t test instead of analysis of variance for repeated measures was used for assessing within group treatment effect. Compared with pre-treatment values, there was no difference in 15-F_2t_-isoprostane concentrations in EBC after 8-week treatment with vitamin E 400 mg once daily indicating that doubling the daily dose of vitamin E (400 UI vs. 200 UI mg once daily) has no effect on oxidative stress as reflected by EBC 15-F_2t_-isoprostane (**Table 2**).

There was no between-group difference in post-treatment 15-F_2t_-isoprostane concentrations in EBC (group A, 314.9 ± 131.7 pg/ml, mean ± SD; group B, 343.1 ± 243.9 pg/ml, *P* = 0.63).

### Measurement of 15-F_2t_-Isoprostane in Serum

In group A, 65 serum samples were analyzed. Thirty-one samples were missing. At least one pre-treatment and post-treatment serum sample were collected from 17 out of 24 patients with CF. Serum 15-F_2t_-isoprostane concentrations were detected in all samples. Data were normally distributed after log transformation. 15-F_2t_-isoprostane concentrations in serum ranged from 10.0 to 1600 pg/ml. Due to missing samples, within group comparison was limited to the 17 CF patients who had both pre-treatment (visit 2) and post-treatment (visit 3) serum samples. Paired *t*-test was used for assessing within group treatment effect. Compared with pre-treatment values, there was no difference in serum 15-F_2t_-isoprostane concentrations (*P* = 0.14) after 8-week treatment with a combination of a higher dose of oral vitamin E (400 UI/daily) and azithromycin (250 or 500 mg once daily depending on body weight) indicating no additional effect on oxidative stress as reflected by serum 15-F_2t_-isoprostane (**Table 3**).

**Table 3:** 15-F_2t_-Isoprostane concentrations in serum in groups A and B^∗^.

Group A (*n* = 24)^#^		
	
	Visit 2 (pre-treatment)	Visit 3 (post-treatment)	*P*-value
15-F_2t_-IsoP (pg/ml)	355.3 ± 410.2 (*n* = 17)^§^	578.2 ± 539.0 (*n* = 17)^§^	0.14

**Group B (*n* = 21)^#^**		
	
	**Visit 2 (pre-treatment) (*n* = 15)^§^**	**Visit 3 (post-treatment) (*n* = 15)^§^**	***P*-value**

15-F_2t_-IsoP (pg/ml)	350.0 ± 261.3 (*n* = 15)	416.0 ± 553.9 (*n* = 15)	0.63

*^∗^Data are expressed as mean ± SD as they were normally distributed after log transformation. Due to missing samples, within group comparison was limited to the CF patients who had both pre-treatment (visit 2) and post-treatment (visit 3) serum samples. Paired t test was used to compare pre-treatment and post-treatment values. Between-group pre-treatment values, assessed by unpaired t-test, were similar (P = 0.97). Between-group post-treatment values, assessed by unpaired t-test, were similar (P = 0.41). Significance was defined as a value of P < 0.05. ^#^Number of CF subjects included in the study group. ^§^ Number of CF patients who had both a pre-treatment and a post-treatment sample. 15-F_*2t*_-IsoP, 15-F_*2t*_-isoprostane.*

In group B, 54 serum samples were analyzed. Thirty samples were missing. At least one pre-treatment and post-treatment serum sample were collected from 15 out of 21 patients with CF. Serum 15-F_2t_-isoprostane concentrations were detected in all samples. Data were normally distributed after log transformation. 15-F_2t_-Isoprostane concentrations in serum ranged from 30.0 to 2210.0 pg/ml. Due to missing samples, within group comparison was limited to the 15 CF patients who had both pre-treatment (visit 2) and post-treatment (visit 3) serum samples. Paired *t*-test was used for assessing within group treatment effect. Compared with pre-treatment values, there was no difference in 15-F_2t_-isoprostane concentrations in serum after 8-week treatment with vitamin E 400 UI once daily indicating that doubling the daily dose of vitamin E (400 UI vs. 200 UI mg once daily) has no effect on oxidative stress as reflected by serum 15-F_2t_-isoprostane (**Table 3**).

Pre-treatment serum 15-F_2t_-isoprostane values in study groups were similar (group A, 355.3 ± 410.2 pg/ml, mean ± SD; group B, 350.0 ± 261.3 pg/ml, *P* = 0.97).

There was no between group difference in post-treatment 15-F_2t_-isoprostane concentrations in serum (group A, 578.2 ± 539.0 pg/ml, mean ± SD; group B, 416.0 ± 553.9 pg/ml, *P* = 0.41).

### Measurement of Plasma Vitamin E

In both study groups, post-treatment plasma vitamin E concentrations [group A: 1140 (877–1615) μg/100 ml (median and interquartile range), *P* = 0.02; group B: 1193 (898–1436) μg/100 ml, *P* = 0.03] were increased compared with pre-treatment plasma vitamin E concentrations [group A: 994 (853–1111) μg/100 ml; group B: 853 (740–1104) μg/100 ml].

### Pulmonary Function Testing

There was no missing data. Spirometry was performed in all study subjects at all visits. Pre-treatment absolute FEV_1_ values (*P* = 0.07), FEV_1_% predicted values (*P* = 0.74), absolute FVC values (*P* = 0.93), FVC% predicted values (*P* = 0.13), and FEV_1_/FVC% (*P* = 0.88) in study groups were similar (**Table 4**).

**Table 4:** Lung function tests in groups A and B^∗^.

Group A (*n* = 24)		
	
	Visit 1 (baseline)	Visit 2 (pre-treatment)	Visit 3 (post-treatment)	Visit 4 (treatment withdrawal)	Overall *P*-value
FEV_1_, L	2.3 ± 0.2	2.3 ± 0.2	2.4 ± 0.2	2.4 ± 0.2	0.80
FEV_1_, % predicted	87.7 ± 5.0	85.7 ± 4.7	84.6 ± 4.7	86.8 ± 5.0	0.52
FVC, L	2.9 ± 0.2	2.9 ± 0.2	2.9 ± 0.2	2.9 ± 0.2	0.99
FVC, % predicted	95.4 ± 4.0	93.6 ± 3.9	94.0 ± 3.7	94.4 ± 4.3	0.81
FEV_1_/FVC, %	81.2 ± 2.4	81.8 ± 2.2	81.4 ± 2.2	80.9 ± 2.4	0.91
**Group B (*n* = 21)**		
	
	**Visit 1 (baseline)**	**Visit 2 (pre-treatment)**	**Visit 3 (post-treatment)**	**Visit 4 (treatment withdrawal)**	**Overall *P*-value**

FEV_1_, L	2.8 ± 0.2	2.8 ± 0.2	2.7 ± 0.2	2.9 ± 0.2	0.15
FEV_1_, % predicted	84.8 ± 4.2	83.5 ± 4.6	82.6 ± 4.4	85.4 ± 4.4	0.61
FVC, L	3.5 ± 0.3	3.4 ± 0.3	3.5 ± 0.3	3.5 ± 0.3	0.77
FVC, % predicted	94.3 ± 3.7	93.1 ± 3.6	92.7 ± 4.0	93.9 ± 3.8	0.82
FEV_1_/FVC, %	81.6 ± 1.9	81.2 ± 1.9	79.2 ± 2.4	82.6 ± 1.7	0.14

*^∗^Data are expressed as mean ± SEM. Repeated one-way ANOVA for repeated measures was used for within-group comparisons. Overall P-value is shown. Significance was defined as a value of P < 0.05. There was no missing data. Spirometry was performed in all study subjects at all visits. FEV_*1*_, forced expiratory volume in one second; FVC, forced vital capacity.*

There was no difference in within group treatment effect of the combination of vitamin E plus azithromycin-group (group A) or vitamin E alone 400 UI once daily (group B) on lung function tests (**Table 4**). There were no between-group differences in post-treatment absolute FEV_1_ values (*P* = 0.19), FEV_1_% predicted values (*P* = 0.76), absolute FVC values (*P* = 0.06), FVC% predicted values (*P* = 0.81), and FEV_1_/FVC% (*P* = 0.51) (**Table 4**).

### Measurement of Serum C-Reactive Protein

There was no missing data. Pre-treatment serum CRP concentrations in study groups were similar (*P* = 0.99) (Supplementary Table S1). There was no within group effect of treatment on serum CRP concentrations (group A: *P* = 0.74; group B: *P* = 0.43) (Supplementary Table S1). There were no between-group differences in post-treatment serum CRP concentrations (*P* = 0.68) (Supplementary Table S1).

### Measurement of C-Reactive Protein in EBC

CRP concentrations in EBC were undetectable.

### NMR-Based Metabolomics of EBC

Ten patients in group A who were on azithromycin and vitamin E and six patients in group B who were on vitamin E alone had the complete set of samples collected at the four study visits. However, due to the limited sample size, we decided to analyze EBC samples obtained from all individual patients. This explains inconsistencies in subject number across visits in both treatment groups. We firstly investigated the “metabolic stability” of CF patients over the 1 week range of baseline (visit 1) and pre-treatment (visit 2) visit (run-in phase) by analyzing the NMR spectra of paired EBC samples. By applying unsupervised PCA (not shown), we could not separate groups A and B. This was confirmed by OPLS-DA, a supervised learning algorithm. Supplementary Figure S1 shows the scores plot of EBC samples obtained from patients with CF included in both treatment groups at visit 1 and visit 2. Although most of the patients’ pairs are clustered around the center of the ellipse, some of them are differently displaced. In particular, pairs 7, 8, and 10 at visit 1 and visit 2 in group A, although dislodged, are nearly close to each other; on the contrary, pairs 1, 13, 17, and 18 in group A and pairs 5 in group B present a stronger separation between visit 1 and visit 2 (Supplementary Figure S1). No between group separation was detected as the quality parameters, which are considered acceptable when values are ≥0.5 (Eriksson et al., 2013), were *R*^2^ (goodness of fit) = 0.21 and *Q*^2^ (goodness of prediction) = 0.19.

The displacement of the above pairs was linked to the presence of a single metabolite (namely, propionate in samples 1 and 10, phenylalanine in sample 17). Limited to pre-treatment vs. post-treatment comparisons, due to the lack of within- and between-group separation at baseline visit (visit 1) and pre-treatment visit (visit 2) (Supplementary Figure S1), data from baseline and pre-treatment visits of both treatment groups (group A, *n* = 20; group B, *n* = 12) were pooled together in order to increase pre-treatment sample size as compared with either treatment in a two time point model (Supplementary Figure S2). Compared with baseline/pre-treatment visit data combining group A and B, the effects of either vitamin E plus azithromycin treatment (Supplementary Figure S2, lower panel) or vitamin E alone (Supplementary Figure S2, upper panel) after 8-week treatment (visit 3) indicated no between visit differences in EBC metabolic profiles. For both plots, *R*^2^ was ≤0.20 and *Q*^2^ was ≤0.22 as quality parameters.

Identical results were obtained by selectively removing outlier samples obtained from patients 1, 10, 17, and 18 (group A) and 5 (group B) from analysis (not shown). Both 8-week treatments do not seem to affect the EBC metabolic distribution in patients with CF.

**FIGURE 2:**
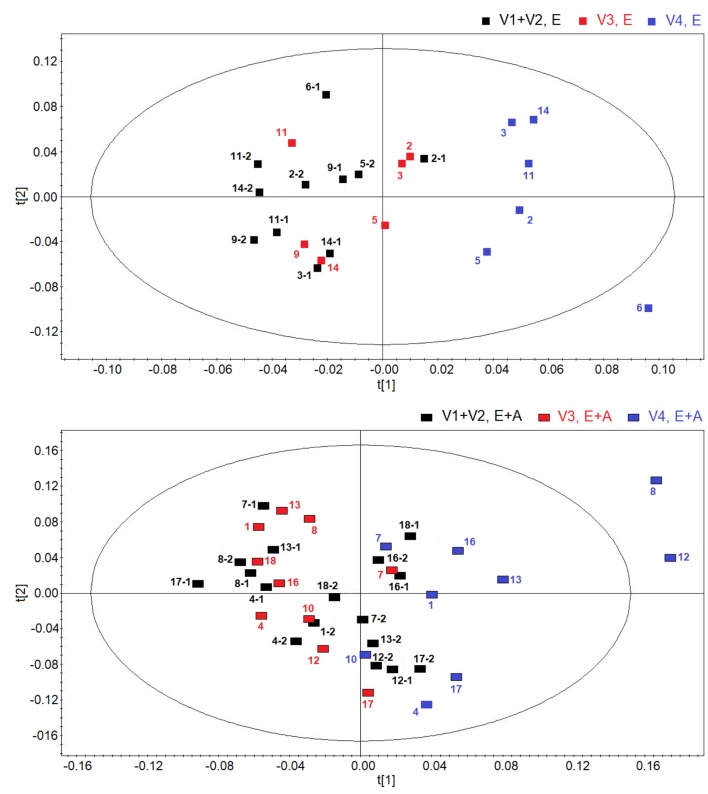
Orthogonal partial least squares projection to latent structures-discriminant analysis (OPLSA-DA) scores plots comparing metabolic distribution in EBC in patients with cystic fibrosis (CF) who were treated with a combination of vitamin E and azithromycin (E + A) (*R*^2^ = 0.73, and *Q*^2^ = 0.64, *P* = 0.028) (black rectangles, *n* = 17; red rectangles, *n* = 10; blue rectangles, *n* = 9) **(lower)** or vitamin E alone (E) (*R*^2^ = 0.80, and *Q*^2^ = 0.69, *P* = 0.031) (black squares, *n* = 11; red squares, *n* = 6; blue squares, *n* = 6) **(upper)** at baseline-pre-treatment visit (visit 1–visit 2, V1–V2), 8 weeks after treatment (visit 3, V3) and 2 weeks after suspension of treatment (visit 4, V4).

**Figure 2** shows score plots comparing metabolic distribution in EBC in group A (lower panel) and group B (upper panel) patients with CF at baseline/pre-treatment (visit 1/visit 2), post-treatment (visit 3) and 2 weeks after suspension of treatment (visit 4) using OPLS-DA supervised clustering in a three-time point model.

Both plots confirm the behavior of post-treatment (visit 3) and correspondent baseline/pre-treatment data set (visit 1/visit 2), while treatment suspension set (visit 4) appears to clusterize in a different region in both groups (group A: *R*^2^ = 0.73, and *Q*^2^ = 0.64, *P* = 0.028; group B, *R*^2^ = 0.80, and *Q*^2^ = 0.69, *P* = 0.031) (**Figure 2**). Considering that EBC samples at visit 4 are collected after 2-week run-out phase, suspension of both treatments induces metabolic changes in patients with CF.

Discrimination between suspension visit and baseline/pre-treatment/post-treatment visit in both group A and group B (**Figure 2**) was found to depend upon the same metabolites, namely ethanol (1.19 and 3.67 ppm), saturated fatty acids (at 1.25 ppm), acetate (at 1.93 ppm), acetoin/acetone (2.23 ppm), and methanol (3.37 ppm). These data suggest that suspension of treatment with vitamin E plus azithromycin or vitamin E alone, after 2 weeks, results in a similar metabolic pattern in EBC. However, quantitative differences in discriminating metabolites, as shown below, could explain why metabolic profiles after treatment suspension in the two study groups are separated.

**FIGURE 3:**
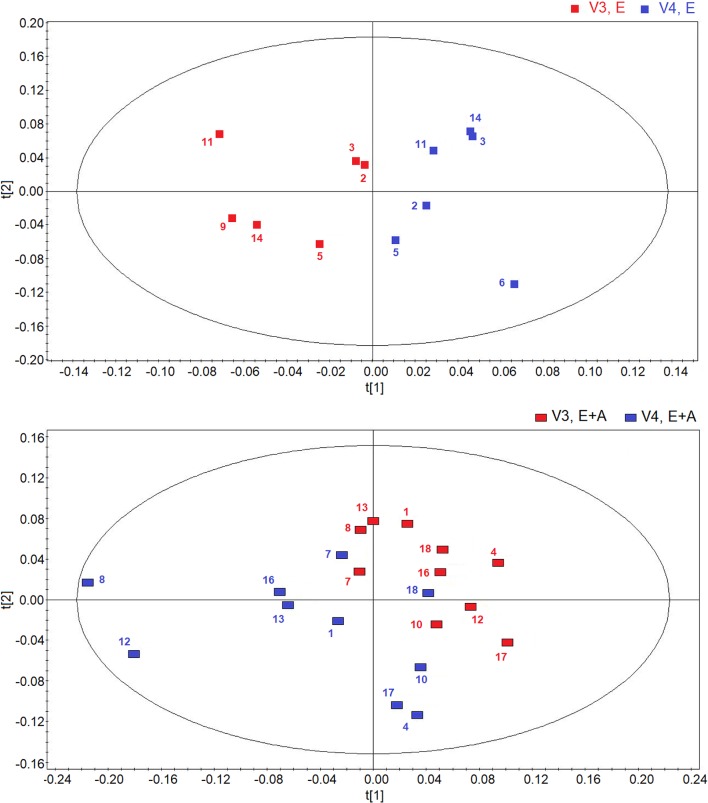
Orthogonal partial least squares projection to latent structures-discriminant analysis (OPLS-DA) scores plots comparing metabolic distribution in EBC in patients with CF who were treated with vitamin E plus azithromycin (E + A) (*R*^2^ = 0.79, and *Q*^2^ = 0.68, *P* = 0.012) (red rectangles, *n* = 10; blue rectangles, *n* = 10) **(lower)** or vitamin E alone (E) (*R*^2^ = 0.83, and *Q*^2^ = 0.71, *P* < 0.001) (red squares, *n* = 6; blue squares, *n* = 6) **(upper)** 8 weeks after treatment (visit 3, V3) and 2 weeks after suspension of treatment (visit 4, V4).

Using OPLS-DA in a two-time point model, we focused on a possible effect of treatment suspension in EBC metabolites. **Figure 3** compares the score plots of both treatments, confirming that in both groups metabolic distribution 2 weeks after treatment withdrawal (run-out phase, visit 4) is different from that observed after 8-week treatment (post-treatment, visit 3) (group A: *R*^2^ = 0.79, and *Q*^2^ = 0.68, *P* = 0.012; group B: *R*^2^ = 0.83, and *Q*^2^ = 0.71, *P* < 0.001), again suggesting an effect of treatment which is detectable after drug suspension.

Interestingly, the metabolites responsible for separation between suspension and post-treatment visit are the same (ethanol, saturated fatty acids, acetate, acetoin/acetone, and methanol).

**FIGURE 4:**
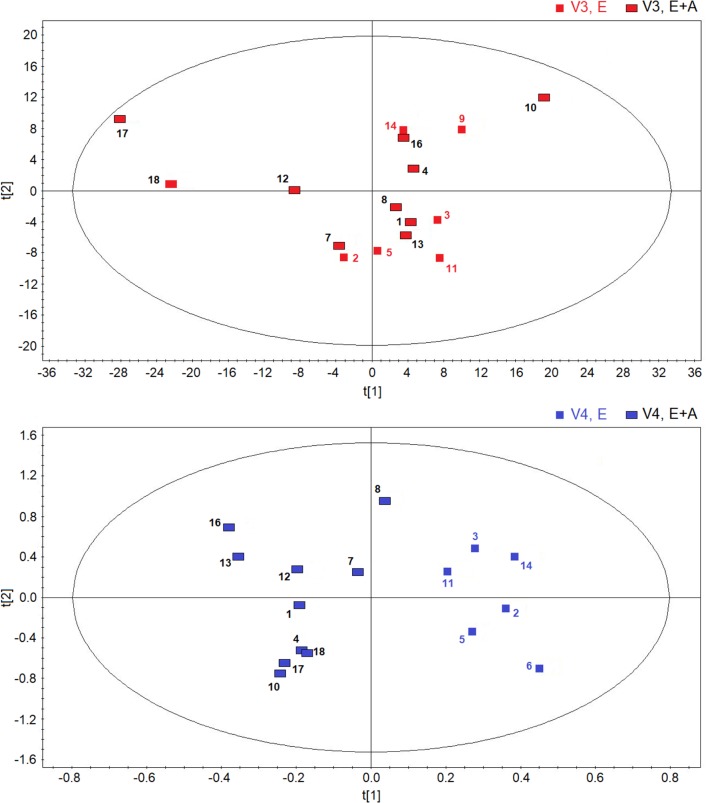
Orthogonal partial least squares projection to latent structures-discriminant analysis scores plots comparing post-treatment (visit 3, V3) metabolic distribution in EBC in patients with CF who were treated with a combination of vitamin E plus azithromycin (E + A) (red rectangles, *n* = 10) or vitamin E alone (red squares, *n* = 6) for 8 weeks (*R*^2^ = 0.66, and *Q*^2^ = 0.62, *P* = 0.032) **(upper)**. OPLS-DA scores plot comparing EBC metabolic profiles in patients with CF who were treated with a combination of vitamin E and azithromycin (E + A) (blue rectangles, *n* = 10) or vitamin E alone (E) (blue squares, *n* = 6) 2 weeks after treatment suspension (visit 4, V4) (*R*^2^ = 0.85, and *Q*^2^ = 0.73, *P* = 0.001) **(lower)**.

However, between group comparisons (group A vs. group B) of post-treatment metabolic distribution (visit 3) shows that both treatments are separated in two defined regions (*R*^2^ = 0.66, and *Q*^2^ = 0.62, *P* = 0.032) (**Figure 4**, upper panel), suggesting that the two treatments are different from each other, but that metabolic differences are not striking with respect to corresponding pre-treatment metabolic distribution (visit 2) (Supplementary Figure S2). A better classification is obtained when between group EBC metabolic profiles at run-out visit (visit 4) are compared (*R*^2^ = 0.85, and *Q*^2^ = 0.73, *P* = 0.001) (**Figure 4**, lower panel). For both comparisons (post-treatment visit, group A vs. post-treatment visit, group B; suspension visit, group, A vs. suspension treatment group B), we identified the same discriminating metabolites as observed in within-group comparisons shown in **Figure 2**. Evaluation of their concentrations indicates that quantitative differences in EBC metabolites explain the observed within- and between-group discrimination. As an example, **Table 5** reports mean EBC metabolite concentrations measured in group A and group B patients with CF at treatment withdrawal visit (visit 4). Interestingly, mean differences in all discriminating metabolites are statistically significant (*P* < 0.05).

**Table 5:** Concentrations of EBC metabolites responsible for classification between group A (vitamin E plus azithromycin) and group B (vitamin E alone) CF patients 2 weeks after treatment withdrawal (run-out phase, visit 4)^∗^.

	Group A (vitamin E plus azithromycin)	Group B (vitamin E)	*P*-value
	(*n* = 10)	(*n* = 6)	
EBC metabolite (μM)			
Acetate	9.78 ± 2.99	6.65 ± 2.07	0.023
Acetoin/acetone	13.48 ± 4.77	19.31 ± 6.43	0.042
Ethanol	10.23 ± 3.92	15.60 ± 5.29	0.015
Methanol	4.89 ± 2.10	2.17 ± 0.91	0.010
Saturated fatty acids	17.71 ± 6.83	10.91 ± 3.28	0.041

*^∗^NMR signals were integrated and referred to the internal TSP signal of known concentration (100 μM). EBC metabolite concentrations are expressed as mean ± SD. P < 0.05 is considered statistically significant. CF, cystic fibrosis; EBC, exhaled breath condensate; NMR, nuclear magnetic resonance; TSP, sodium trimethylsilylpropionate.*

Taken together, EBC metabolomics data suggest that doubling the dose of vitamin E with or without azithromycin for 8 weeks does not substantially change EBC metabolites in patients with stable CF compared with metabolic distribution observed at baseline/pre-treatment visit. However, due to quantitative differences in their EBC concentrations, within-group comparisons show that treatment suspension is associated with a different EBC metabolite distribution in both groups, whereas between-group comparisons show that EBC metabolite profiles in the two treatment groups are different after 8 weeks of treatment (**Figure 4**, upper panel) and, to a greater extent, 2 weeks after treatment withdrawal (**Figure 4**, lower panel). These findings might suggest that (1) unlike treatment suspension, the effect of both treatment themselves is not strong enough to cause post-treatment EBC metabolic changes, possibly due to within group inter-individual variability; (2) compared with vitamin E alone, adding azithromycin changes EBC metabolomic profiles in CF patients as reflected by between-group metabolite distribution post-treatment and after treatment suspension; (3) a build-up effect might be present in CF patients 2 weeks after vitamin E plus azithromycin and vitamin E treatment withdrawal (run-out phase, visit 4), suggesting longer evaluation for detecting pharmacological effects.

### NMR Metabolomics of Serum

Metabolomic analysis of serum with NMR spectroscopy suggests that EBC is a more specific matrix for the metabolic analysis of these groups of patients with CF.

All models derived from serum spectra have shown a large spectral variability, which appears to be independent from classes, pharmacological treatment, and different visits.

**FIGURE 5:**
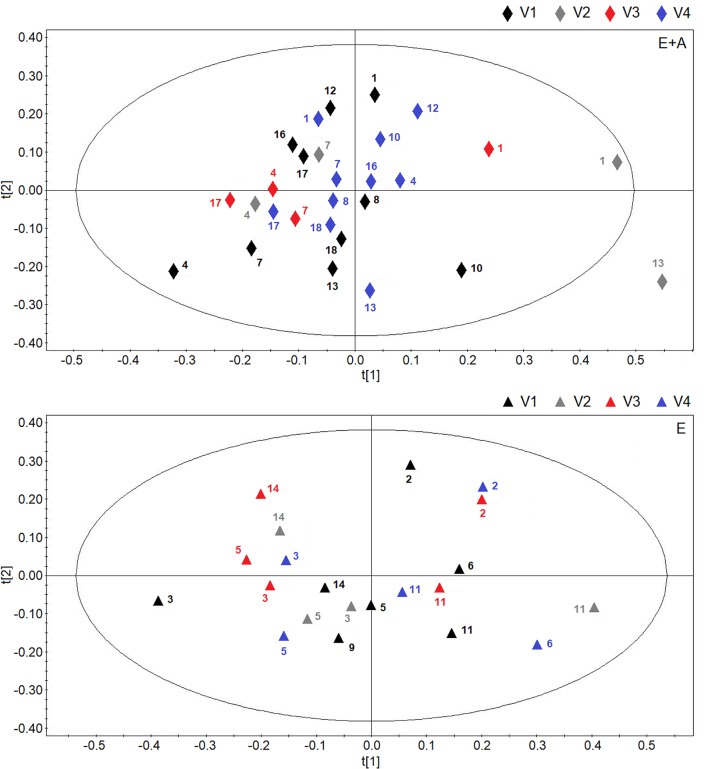
Scores plot obtainedes plot obtained from orthogonal partial least squares projection to latent structures-discriminant analysis analysis of serum NMR spectra obtained from CF patients who were treated with vitamin E plus azithromycin (E + A) (*R*^2^ = 0.16, and *Q*^2^ = 0.20, *P* = 0.44) in the four visits (visit 1, V1, black lozenges, *n* = 10; visit 2, V2, dark gray lozenges, *n* = 4; visit 3, V3, red lozenges, *n* = 4; visit 4, V4, blue lozenges, *n* = 10) **(upper)** and CF patients who were treated with vitamin E alone (*R*^2^ = 0.19, and *Q*^2^ = 0.18, *P* = 0.56) in the four visits (visit 1, V1, black triangles, *n* = 7; visit 2, V2, dark gray triangles, *n* = 4; visit 3, V3, red triangles, *n* = 5; visit 4, V4, blue triangles, *n* = 5) **(lower)**. Numbers refer to patients.

**Figure 5** reports the OPLS-DA scores plots from serum NMR spectra of CF patients who were treated with vitamin E plus azithromycin (group A) (upper panel) or vitamin E alone (group B) (lower panel) in a 4-time point model.

In both scores plots, we did not observe variations due to the temporal evolution of the pharmacological treatment as we obtained very low quality parameters and models with null components.

Likewise, comparisons of groups A and B NMR spectra after 8-week treatment or 2 weeks after treatment suspension, did not show any between group difference in serum metabolites.

To summarize, no within-group or between-group discrimination was observed using NMR-based serum metabolomics suggesting that EBC is a more specific matrix for the metabolic analysis of these groups of patients with CF.

### Correlations

There was no correlation among outcome measures, including metabolomics data, across visits in any study groups.

## Discussion

*In vitro* and *in vivo* experimental studies show that azithromycin reduces oxidative stress by several mechanisms including increased apoptosis and inhibition of oxidant burst, chemotactic responses, and myeloperoxidase production in neutrophils (Culic et al., 2001, 2002; Tamaoki et al., 2004; Tsai and Standiford, 2004; Parnham et al., 2014). These effects might have potential clinical implications due to the pivotal role of neutrophils in the pathophysiology of CF (Cockx et al., 2017; Rada, 2017).

We did not observe significant changes in lung function tests after 8-week treatment with azithromycin possibly due to the short duration of treatment and the fact that less than 50% of CF patients included in our study had chronic *P. aeruginosa* infection. As a matter of fact, improvements in lung function have been reported after 24-week treatment with azithromycin and limited to CF patients with chronic *P. aeruginosa* infection (Saiman et al., 2003). In particular, in this 24-week randomized, placebo-controlled, study including 185 CF patients with chronic *P. aeruginosa* infection, treatment with oral azithromycin at a dose of 250 mg (weight < 40 kg) or 500 mg (weight ≥ 40 kg) once daily 3 days per week (*n* = 87) was associated with improvement in forced expiratory volume in 1 s (FEV_1_) (primary outcome) at study end (0.097 L (*SD*, 0.26) compared with 0.003 L (*SD*, 0.23) in the placebo group (*n* = 98) (mean difference, 0.094 L; 95% confidence interval [CI], 0.023–0.165; *P* = 0.009) (Saiman et al., 2003). Likewise, azithromycin reduced exacerbation rate (secondary outcome) compared with placebo (hazard ratio, 0.65; 95% CI, 0.44–0.95; *P* = 0.03) (Saiman et al., 2003). However, it is unknown whether these clinical effects of azithromycin were due to its potential anti-inflammatory effects as inflammatory markers were not measured in this study. Antibiofilm activities against *P. aeruginosa* at therapeutic concentrations (Li et al., 2017) and effects on bacterial communication (quorum sensing) (Skindersoe et al., 2008) might explain, at least partially, the clinical efficacy of azithromycin reported in this study (Saiman et al., 2003). However, the antibacterial effect of azithromycin is unlikely to explain pulmonary function improvement as *P. aerugino*sa is considered naturally resistant to macrolides (Girard et al., 1987) and no differences in the airway colonization by *P. aeruginosa* were observed during the treatment with azithromycin for more than 12 months in a retrospective study in CF patients (Samson et al., 2016). On the other hand, a potential concern on azithromycin, that is treatment-emergent respiratory pathogens, seems to be unjustified. A recent retrospective study reports a lower risk of acquiring several CF-related respiratory pathogens including methicillin-resistant *S. aureus*, non-tuberculous mycobacteria, and *Burkholderia cepacia* complex in chronic azithromycin users compared with non-users, whereas the risk for acquisition of other pathogens, including *P. aeruginosa*, in the two groups was similar (Cogen et al., 2018).

Unlike a previous study in CF patients who were infected with *P. aeruginosa* (Saiman et al., 2003), a 24-week, randomized, placebo-controlled, study aiming at assessing the effect of oral azithromycin [250 mg (weight 18-35.9 kg) or 500 mg (weight ≥ 36 kg)] once daily 3 days per week on FEV_1_ (primary outcome) in 260 patients with CF who were uninfected with *P. aeruginosa* failed to demonstrate post-treatment improvements in FEV_1_ (primary outcome) [mean between group difference 0.02 L (95% CI, -0.05 to 0.08; *P* = 0.61)], whereas exacerbation rate (secondary outcome) was reduced by 50% compared with placebo (95% CI, 31–79%) (Saiman et al., 2010). Treatment with azithromycin was associated with reduction in serum inflammatory markers including absolute and differential white blood cell counts, myeloperoxidase, high-sensitivity CRP, intracellular adhesion molecule 1, interleukin (IL)-6, calprotectin, amyloid A, and granulocyte colony-stimulating factor (Ratjen et al., 2012).

A cross-sectional study was unable to show anti-inflammatory effects of azithromycin (Shmarina et al., 2017). Plasma tumor necrosis factor-α concentrations were higher, whereas plasma concentrations of IL-10, which inhibits synthesis of pro-inflammatory cytokines, were lower in CF patients who received basic therapy along with azithromycin (*n* = 59) compared with CF patients who received basic therapy only (*n* = 102) (Shmarina et al., 2017).

However, measuring serum inflammatory markers could not be the best strategy for assessing pulmonary pathophysiology as they reflect systemic rather respiratory inflammation (Ratjen et al., 2012).

In patients with CF, the effects of azithromycin on lung oxidative stress, which represents one aspect of the inflammatory process, are mostly unknown.

In our pilot, proof-of-concept, randomized, open-label, parallel group, pharmacological study, treatment with either oral azithromycin plus vitamin E 400 UI once daily or oral vitamin E alone 400 UI once daily for 8 weeks was not associated with a reduction in EBC 15-F_2t_-isoprostane compared with baseline values in patients with CF who were on vitamin E at a constant dose of 200 mg UI once daily for at least 12 weeks. Post-treatment EBC 15-F_2t_-isoprostane concentrations in the two study groups were similar. These findings suggest that azithromycin as add-on treatment with doubling the dose of oral vitamin E or doubling the dose of oral vitamin E only has no antioxidant effect in patients with CF as reflected by EBC 15-F_2t_-isoprostane, a marker of lipid peroxidation. Reasons for the lack of evidence of treatment antioxidant effect in our study might rely on the fact that assessment of oxidative stress was limited to 15-F_2t_-isoprostane, a marker of lipid peroxidation which is only one aspect of the complex oxidative stress process, and the relatively short duration of treatment (8-weeks).

However, results from a cross-sectional study, in which several markers of oxidative stress were measured and average duration of treatment with azithromycin was 38 ± 22 months, are not consistent with this explanation of our findings as levels of serum 15-F_2t_-isoprostane, total antioxidant capacity, superoxide dismutase activity, catalase activity, glutathione peroxidase activity, and thiobarbituric acid reactive substances, in CF patients who had been taking azithromycin (*n* = 23) and in CF patients who had not been receiving azithromycin (*n* = 13) were similar (Olveira et al., 2017). Nevertheless, differences in study design (cross-sectional vs. interventional) and biological matrices in which markers were measured (serum vs. EBC) make it difficult comparing this study and our study. Although doubling the dose of oral vitamin E resulted in increased systemic exposure after 8-week treatment as shown by its plasma concentrations, the lack of antioxidant effect of oral vitamin E 400 UI once daily observed in our study might also reflect a dose which is still insufficient to reach effective antioxidant plasma levels (Keljo et al., 2000). On the other hand, fat-soluble vitamins including vitamin E are routinely supplemented in CF to prevent deficiencies associated with fat malabsorption (Okebukola et al., 2017), whereas their therapeutic use as antioxidants is limited, also in view of the fact that antioxidant doses of vitamin E in CF patients are not established (Ciofu and Lykkesfeldt, 2014) due to the incomplete knowledge of its clinical pharmacology. Although it did not meet the primary outcome of 15% improvement in FEV_1_% of predicted value, a 12-month study showed that treatment with inhaled glutathione improved FEV_1_ values over baseline at 3, 6, and 9 months therapy in CF patients with moderate lung disease (Calabrese et al., 2015). Further studies on the potential therapeutic effects of antioxidant treatment in CF are warranted.

Metabolomic analysis of EBC with NMR spectroscopy shows that most of the samples present a good intra-, intergroup, and intrasubject metabolic stability as observed by comparing baseline and pre-treatment visits, while others have a good intrasubject metabolic stability, but show inter and intragroup differences.

The fact that some paired EBC samples show variability over the 1 week run-in phase might indicate that some study patients present metabolic changes that are not detectable by clinical and functional parameters, since subjects were all considered to be stable over the 1-week run-in period.

Doubling the dose of vitamin E with or without azithromycin for 8 weeks does not substantially change EBC metabolites in patients with stable CF compared with metabolic distribution observed at baseline/pre-treatment visit. By contrast, EBC metabolic profiles at post-treatment and withdrawal visit are separated in both study groups. These findings might suggest that suspension of treatment is associated with a more pronounced effect on EBC metabolites than treatment itself whose effect might be leveled out by within group intersubject variability.

Furthermore, the possibility of a build-up effect observed after treatment withdrawal should be carefully evaluated.

Interestingly, between group comparisons show EBC metabolic differences at post-treatment and, more markedly, at withdrawal visit suggesting an effect of azithromycin on metabolomics profiles in CF patients. Quantitative differences in discriminating EBC metabolites, including ethanol, saturated fatty acids, acetate, acetoin/acetone, and methanol, are likely to explain between group differences in metabolic distribution observed after treatment and treatment suspension. Of note, in a previous study, we reported that three of these metabolites, namely ethanol, acetate and methanol, were able to discriminate between patients with stable and unstable CF in a four selected EBC metabolite-based classification model (Montuschi et al., 2012).

We were not able to study a direct effect of azithromycin as we did not include a group treated with azithromycin alone because suspension of treatment with vitamin E, which is standard of care in CF patients, for 10 weeks would not have been acceptable.

Taken together, the analysis of the above preliminary data suggests that NMR-based metabolomics of EBC might be used to follow the “metabolic stability” of a set of CF patients and the “metabolic effect” of suspension of different pharmacological treatments.

Limitations of our study include the open label design, the lack of an azithromycin alone arm, which was excluded for ethical reasons, the use of a single marker for assessing of oxidative stress, which is a complex pathophysiological process, the numerous missing EBC and serum samples which precluded the assessment of treatment effect on 15-F_2t_-isoprostane across all visits. The latter also explains the small number of CF patients on which the EBC metabolomics models are based.

Strengths of our study include the prospective design, the choice of 15-F_2t_-isoprostane, a robust and well established marker of lipid peroxidation, as a primary outcome, the use of EBC NMR-based metabolomics which is a powerful and validated tool for exploring the pathophysiology and the effects of pharmacological treatment at a molecular level in patients with CF.

## Conclusion

Our proof-of-concept, pilot study was unable to show antioxidant effect of azithromycin as add-on treatment with doubling the dose of oral vitamin E as reflected by EBC and serum 15-F_2t_-isoprostane concentrations. The metabolomics analysis of EBC with NMR spectroscopy shows that suspension of both azithromycin plus vitamin E and vitamin E alone has a striking effect on metabolic profiles in EBC. Add-on therapy with azithromycin is not associated with EBC metabolite changes, but EBC metabolite distribution after treatment and 2 weeks after treatment suspension in the two treatment groups is different, suggesting a possible effect of azithromycin. Quantitative differences in EBC concentrations of ethanol, saturated fatty acids, acetate, acetoin/acetone, and methanol are likely responsible for these differences. Due to the small sample size, these preliminary results require further investigation in larger studies in which metabolomics models should be validated with a training and testing approach.

The pathophysiological and therapeutic implications of EBC metabolite changes in patients with stable CF observed in our study are unknown and require further research.

Our preliminary data suggest that NMR-based metabolomics of EBC might be used for assessing the effects of withdrawal of pharmacological treatments in stable CF patients.

## Author Contributions

PM was responsible for study planning, study design, measurement of 15-F2t-isoprostane in EBC and serum, data analysis, data interpretation, and manuscript writing. VL was responsible for clinical trial, patient recruitment, data collection, spirometry, and manuscript revision. DP was responsible for EBC and serum NM spectroscopy, multivariate data analysis, data interpretation, and manuscript revision. EM was responsible for patient recruitment, clinical trial, data collection, spirometry, and manuscript revision. RS was responsible for measurement of 15-F_2t_-isoprostane in EBC and serum. NM and GS was responsible for data analysis, data interpretation, and manuscript revision. DM was responsible for EBC and serum NM spectroscopy, multivariate data analysis, data interpretation, and manuscript revision. FM was responsible for patient recruitment, clinical trial, data collection, and spirometry. AM was responsible for EBC and serum NM spectroscopy, multivariate data analysis, data interpretation, and manuscript writing.

## Conflict of Interest Statement

The authors declare that the research was conducted in the absence of any commercial or financial relationships that could be construed as a potential conflict of interest.
