# Supplementary material for: Metabolomic Analysis by Nuclear Magnetic Resonance Spectroscopy as a New Approach to Understanding Inflammation and Monitoring of Pharmacological Therapy in Children and Young Adults With Cystic Fibrosis
Source: Front Pharmacol. 2018 Jun 18;9:595. doi: 10.3389/fphar.2018.00595 (PMC6015879; doi:10.3389/fphar.2018.00595)
Supplement: Supplementary file 1 [file Table_1.DOCX]

Table S1. Serum CRP concentrations in groups A and B*

| Group A (n = 24) | | | | | |
| --- | --- | --- | --- | --- | --- |
|  | Visit 1  (baseline) | Visit 2  (pre-treatment) | Visit 3  (post-treatment) | Visit 4  (treatment withdrawal) | Overall P value |
| CRP (mg/L) | 0.11 (0.05-0.39) | 0.09 (0.05-0.50) | 0.13 (0.05-0.57) | 0.15 (0.05-0.65) | 0.74 |
|  | Visit 1  (baseline) | Visit 2  (pre-treatment) | Visit 3  (post-treatment) | Visit 4  (treatment withdrawal) | Overall P value |
| Group B (n = 21) | | | | | |
|  | Visit 1  (baseline) | Visit 2  (pre-treatment) | Visit 3  (post-treatment) | Visit 4  (treatment withdrawal) | Overall P value |
| CRP (mg/L) | 0.10 (0.05-0.45) | 0.06 (0.03-0.85) | 0.05 (0.03-0.62) | 0.05 (0.03-0.50) | 0.43 |

*Data are expressed as median and interquartile range. Friedman test was used for within group comparisons. Overall P value is shown. Significance was defined as a value of P < 0.05. There was no missing data. Abbreviation: CRP, C reactive protein.
