# Supplementary material for: Metabolomic Analysis by Nuclear Magnetic Resonance Spectroscopy as a New Approach to Understanding Inflammation and Monitoring of Pharmacological Therapy in Children and Young Adults With Cystic Fibrosis
Source: Front Pharmacol. 2018 Jun 18;9:595. doi: 10.3389/fphar.2018.00595 (PMC6015879; doi:10.3389/fphar.2018.00595)
Supplement: Supplementary file 2 [file Presentation_1.pdf]

Figure legends

Figure S1. Orthogonal partial least squares projection to latent structures-discriminant analysis (OPLSA-DA) scores plot of patients' stability. Patients with cystic fibrosis (CF) were checked for their stability during baseline visit (visit 1) and pre-treatment visit (visit 2) (run in phase, one week interval). Labeling refer to patient (first number), also classifying the visit (1 or 2). Dark-gray rectangles identify samples obtained from patients with CF who were included in group A (treatment with vitamin E plus azithromycin, E + A, n = 20), whereas light-grey squares identify samples obtained from patients with CF who were included in group B (treatment with vitamin E alone, E, n = 12). The goodness of fit (R^2) and the goodness of prediction (Q^2) were 0.21 and 0.19, respectively.

Figure S2. Orthogonal partial least squares projection to latent structures-discriminant analysis (OPLSA-DA) scores plots comparing the effect of vitamin E plus azithromycin (E + A) (group A) (red rectangles, n = 10; black squares, n = 32) (lower panel) and vitamin E alone (E) (group B) (red squares, n = 6; black squares, n = 32) (upper panel) on EBC metabolites after eight-week treatment (visit 3, V3) compared to baseline-pre-treatment metabolic distribution (visit 1-visit 2, V1-V2). For both plots, R^2 was ≤ 0.20 and Q^2 was ≤ 0.22 as quality parameters. Limited to pre-treatment vs. post-treatment comparisons, due to the lack of within- and between-group separation at baseline visit (visit 1) and pre-treatment visit (visit 2) (Figure 2), data from baseline and pre-treatment visits of both treatment groups (group A, n = 20; group B, n = 12) were pooled together in order to increase pre-treatment sample size as compared with either treatment in a two time point model.
