# Supplementary figures and images for: Metabolomic Analysis by Nuclear Magnetic Resonance Spectroscopy as a New Approach to Understanding Inflammation and Monitoring of Pharmacological Therapy in Children and Young Adults With Cystic Fibrosis
Source: Front Pharmacol. 2018 Jun 18;9:595. doi: 10.3389/fphar.2018.00595 (PMC6015879; doi:10.3389/fphar.2018.00595)

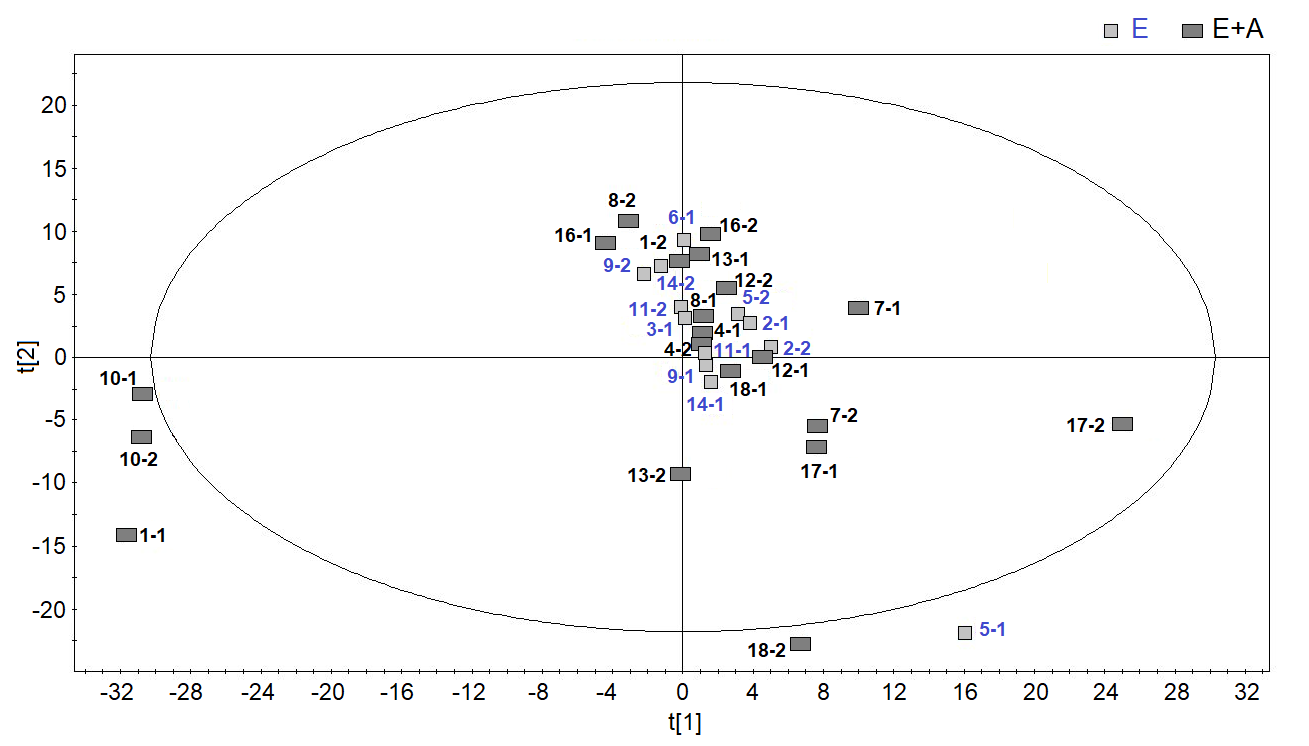

Supplement: Supplementary file 3 [file Image_1.TIFF]

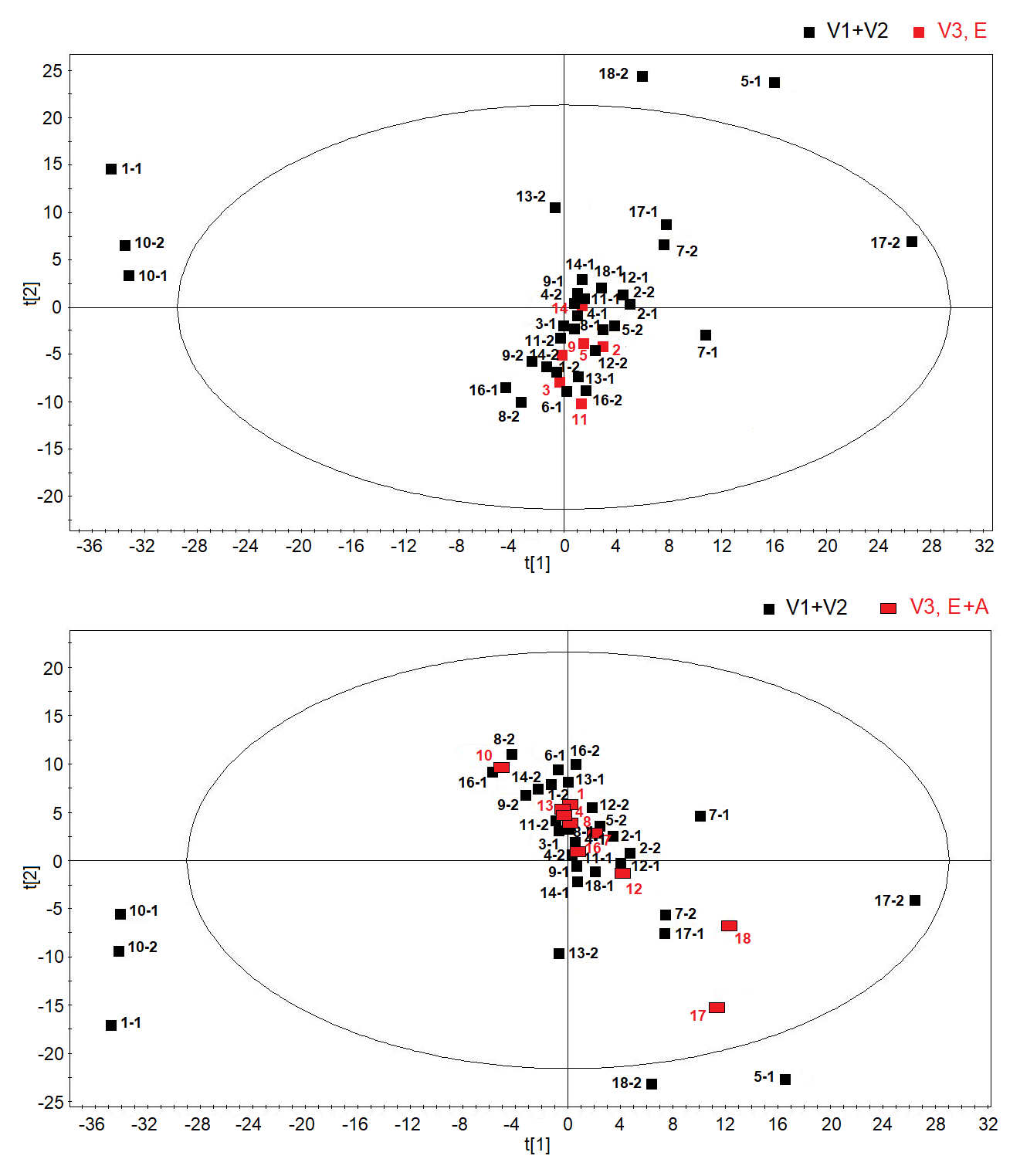

Supplement: Supplementary file 4 [file Image_2.TIFF]
